# RNA editing blood biomarkers for predicting mood alterations in HCV patients

**DOI:** 10.1007/s13365-019-00772-9

**Published:** 2019-07-22

**Authors:** N. Salvetat, S. Van der Laan, B. Vire, F. Chimienti, S. Cleophax, J. P. Bronowicki, M. Doffoel, M. Bourlière, R. Schwan, J. P. Lang, J. F. Pujol, D. Weissmann

**Affiliations:** 1 ALCEDIAG, Sys2Diag / CNRS UMR9005, Parc Euromédecine Cap Delta, 1682 Rue de la Valsière, 34184 Montpellier Cedex 4, France; 2 Biocortech, rue de la Croix Jarry, 75013 Paris, France; 3 Present Address: Etablissement Français du Sang, 2 avenue Ile de France, 95300 Pontoise, France; 4 Hepato-Gastroenterology, Hopital Brabois- CHU Nancy, 54511 Vandoeuvre-les-Nancy, France; 5 Université de Strasbourg, Hopital Universitaire de Strasbourg, 67000 Strasbourg, France; 6 Hepato-Gastroenterology, Hopital Saint Joseph, 13285 Marseille, France; 7 Present Address: Les Toises, Centre de psychiatrie et psychothérapie, Lausanne, Switzerland

**Keywords:** A-to-I RNA editing, Depression, Inflammation, Epitranscriptomic biomarkers, Interferon alpha, Chronic hepatitis C virus, PDE8A, ADAR

## Abstract

Treatment-emergent depression is a common complication in patients with chronic hepatitis C virus (HCV) infection undergoing antiviral combination therapy with IFN-α and ribavirin. It has recently been shown that changes in A-to-I RNA editing rates are associated with various pathologies such as inflammatory disorders, depression and suicide. Interestingly, IFN-α induces gene expression of the RNA editing enzyme ADAR1-1 (ADAR1a-p150) and alters overall RNA editing activity. In this study, we took advantage of the high prevalence of pharmacologically induced depression in patients treated with IFN-α and ribavirin to test the interest of RNA editing–related biomarkers in white blood cells of patients. In this 16-week longitudinal study, a small cohort of patients was clinically evaluated using standard assessment methods prior to and during antiviral therapy and blood samples were collected to analyse RNA editing modifications. A-I RNA editing activity on the phosphodiesterase 8A (PDE8A) gene, a previously identified RNA editing hotspot in the context of lupus erythematosus, was quantified by using an ultra-deep next-generation sequencing approach. We also monitored gene expression levels of the ADAR enzymes and the PDE8A gene during treatment by qPCR. As expected, psychiatric evaluation could track treatment-emergent depression, which occurred in 30% of HCV patients. We show that PDE8A RNA editing is increased in all patients following interferon treatment, but differently in 30% of patients. This effect was mimicked in a cellular model using SHSY-5Y neuroblastoma cells. By combining the data of A-I RNA editing and gene expression, we generated an algorithm that allowed discrimination between the group of patients who developed a treatment-emergent depression and those who did not. The current model of drug-induced depression identified A-I RNA editing biomarkers as useful tools for the identification of individuals at risk of developing depression in an objective, quantifiable biological blood test.

**Electronic supplementary material:**

The online version of this article (10.1007/s13365-019-00772-9) contains supplementary material, which is available to authorized users.

## Introduction

Catalytic deamination of adenosine (A) to inosine (I) on RNA molecules is a fundamental cellular mechanism that directly impacts expression and function of a large set of proteins. Deamination is brought about by the action of specific editing enzymes, termed ADAR (adenosine deaminase acting on RNA). In vertebrates, the ADAR family is composed of three different genes, namely ADAR1, ADAR2 and ADAR3. All ADARs share common modular organisation of domain structures that comprise a double-stranded RNA-binding domain (dsRBD) necessary for target binding and a catalytic domain conveying deaminase activity (Gallo et al. 2017). The formation of pre-mRNA double-stranded stem loops during transcription constitutes the prime substrate of the ADAR enzymes. During the editing process, adenosines that reside in the coding sequence (e.g. 5-HT2c receptor, glutamate receptor) or in non-repetitive intronic sequences (e.g. phosphodiesterase subtype 8A/PDE8A) are deaminated. While alterations in the coding sequence may lead to a change in amino acid composition and function of the translated protein (Berg et al. 2001; Burns et al. 1997), the editing process of the intronic sequences has been proposed to influence both RNA splicing pattern and formation of different isoforms of microRNAs, known to actively modulate gene expression (Kawahara et al. 2008; Solomon et al. 2013; Wulff and Nishikura 2012).

A growing body of evidence in the literature has shown that deregulation of RNA editing activity is tightly associated with numerous pathologies such as cancer (Chen et al. 2013; Chen et al. 2017; Crews et al. 2015; Fumagalli et al. 2015; Gallo and Galardi 2008) and neuropsychiatric and/or inflammatory disorders (Di Narzo et al. 2014; Dracheva et al. 2008; Orlowski et al. 2008; Weissmann et al. 2016). Previous studies have shown that the serotonin 2c receptor (5-HT2cR) pre-mRNA editing is modified in mental disorders and has been associated with suicide and schizophrenia (Kubota-Sakashita et al. 2014; Lyddon et al. 2013). In the context of suicide, we and others have observed a specific 5-HT2cR editing signature in post-mortem brain samples of suicide victims as compared with non-suicidal and otherwise normal individuals (Di Narzo et al. 2014; Dracheva et al. 2008; Gurevich et al. 2002; Weissmann et al. 2016). Interestingly, antidepressant and antipsychotic use was also shown to alter the 5-HT2c receptor pre-mRNA editing in specific area of the brain (Englander et al. 2005). Combined observations of pathological and pharmacological alterations of RNA editing activity on the 5-HT2cR in the brain laid the foundation for the development of an in vitro high-throughput neurotoxicity test allowing identification of compounds and drugs that act on RNA editing (van der Laan et al. 2017). The neurotoxicity test is based on accurate quantification of RNA editing changes of the 5-HT2cR in a well-defined human neuroblastoma cell line (Cavarec et al. 2013). Interestingly in this model, interferon alpha (IFN-α) treatment, a widely used antiviral therapy recognised to induce mood alterations in a large proportion of patients (Schaefer et al. 2013), dose-dependently alters the mRNA editing profile of 5-HT2cR via transcriptional activation of the ADAR1-1 (ADAR1a-p150) isoform (Cavarec et al. 2013).

The link between altered inflammation and neuropsychiatric disorders has been proposed for a long date, and compelling evidence support the tight interplay between the immune system and mood disorders (for review, see Wohleb et al. 2016). In a blinded study that directly confirmed the causality between the two systems, healthy individuals were administered a low dose of lipopolysaccharide (LPS, bacterial endotoxin) or a placebo treatment. Interestingly, significant increase in depressive symptoms was observed in the LPS-injected subjects as compared with controls (Reichenberg et al. 2001), directly providing a link between stimulated immune response and mood alteration. The exact mechanism by which the link is embodied remains elusive although various mechanisms have been proposed. In line with this notion, type I interferon signalling was recently shown to be altered in blood cells of patients suffering from severe recurrent depression (Mostafavi et al. 2014). The relationship between mood disorders and interferon type I is further substantiated by numerous case reports that extensively document the altered mood of patients undergoing interferon treatment in the context of various disorders such as multiple sclerosis (Lacy et al. 2013), cancer and/or hepatitis C infection (Bonaccorso et al. 2002; Fragoso et al. 2010). Moreover, upon arrest of therapy, full remission of the psychiatric side effects induced by the IFN treatment has been observed in a subgroup of patients underpinning the pharmacological causality.

The phosphodiesterase 8A (PDE8A) transcript, which is a target of ADARs, is expressed both in brain and in blood tissues and edited in humans and pre-human species. Different studies have indicated the involvement of the 15q chromosomic locus containing PDE8A gene in major depressive disorder and recurrent depression (Holmans et al. 2004). Significant linkage to major depressive disorder (MDD) on chromosome 15q25-q26 was subsequently confirmed after fine mapping with single nucleotide polymorphism markers (Levinson et al. 2007). A specific non-repetitive intronic region of the PDE8A gene is targeted by ADARs and has recently been shown to be dysregulated in systemic autoimmune lupus erythematosus disorder. Orlowski and collaborators identified two hot spots for A-to-I editing in the PDE8A gene transcripts in T lymphocytes (Orlowski et al. 2008). We recently identified region-specific alterations of RNA editing in PDE8A mRNA in the brain, which could discriminate between controls and depressed suicide decedents (Chimienti et al. 2019). These studies paved the way for the development of tests to predict depression through the measurement of PDE8A editing and ADAR activity on peripheral blood in depressed patients.

In this study, we took advantage of the well-defined paradigm consisting of hepatitis C patients that undergo antiviral therapy to tightly monitor mood alterations and RNA editing biomarkers. Specifically, we aimed to identify IFN-induced depression signature by measuring expression of ADAR1-1 (ADAR1a-p150), ADAR1-5 (ADAR1b-p110), ADAR2 and PDE8A mRNA editing profiles in blood samples. We show that a combination of ADAR expression level and specific PDE8A RNA editing profile gene allows discrimination between the group of patients who developed a treatment-emergent depression and those who did not, suggesting our model as a useful tool for the identification of individuals at risk of developing depression during antiviral combination therapy.

## Material and methods

### Human subjects and study design

This study enrolled 10 patients infected with hepatitis C virus (HCV) from three different hospitals in France (Centre Hospitalier Universitaire of Marseille, Nancy and Strasbourg). Eligible participants were adults due to commence combination antiviral therapy with pegylated interferon alpha-2a (IFN-α-2a) and ribavirin for at least 12 weeks; this comprised weekly IFN-α-2a (180 μg) injection and daily ribavirin tablets (1000–1200 mg/day orally for a body weight inferior or superior to 75 kg). Exclusion criteria included the following: age below 18 years, current diagnosis (at baseline) of major depressive disorder. Written informed consent was obtained from all subjects after a detailed discussion on the study aims and requirements. All subjects were able to understand informed consent detailing the research goals and procedure. The demographic data on age and gender were recorded (Table 1); patients did not have history of psychiatric disorders. The study was approved by the local Institutional Review Board, according to the approval requirements and good clinical practice. An initial blood sample was collected 4 weeks prior to antiviral therapy and every 2 weeks during the 12 weeks after onset of treatment (longitudinal analysis).

**Table 1:**
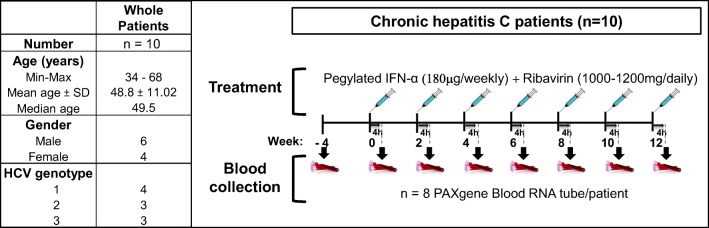
Clinical and virological characteristics of the 10 patients included in the study

### Clinical assessments

Patients were recruited at baseline before starting pegylated interferon alpha-2a and undertook prospective psychiatric evaluations. Psychiatric diagnostics were determined according to the Diagnostic and Statistical Manual of Mental Disorders IV (DSM-IV) and the *International* Classification of Disease-10 Classification of Mental Disorders (ICD-10). The Mini International Neuropsychiatric Interview (MINI) was administered at baseline, to assess current depression or a previous history of depression, and at follow-up assessments for the detection of new-onset cases of depression. The MINI is a structured diagnostic interview for psychiatric disorders according to the DSM-IV (Sheehan et al. 1998). Depression severity was assessed using the Montgomery-Åsberg Depression Rating Scale (MADRS). In addition, severity of manic symptoms using Young Mania Rating Scale (YMRS) and emotional reactivity using Multidimensional Assessment of Thymic States (MAThyS) were also administered both at baseline and at follow-up evaluations. The population was divided into two groups composed of patients having a psychiatric event (combined clinical evaluations) or not. Three patients (30%) developed psychiatric events during the treatment whereas 7 patients (70%) did not develop depression.

### Cell culture, IFN-α treatment, cell lysis and RNA extraction

The SH-SY5Y human neuroblastoma cells were purchased from ECACC (ref 94030304). They were cultured in high-glucose D-MEM with stable glutamine (PAA Laboratories) supplemented with 10% dialysed FCS (PAA Laboratories) and a solution of stabilised Antibiotic-Antimycotic (Sigma-Aldrich). SH-SY5Y cells were plated in 6-well plates at a density of 7.10^5^ cells/well. The next day, culture medium was removed and cells were incubated for 48 h with increasing concentrations of recombinant hIFN-α (1, 10, 100, 1000 and 10,000 IU/ml, *n* = 8 for each concentration, PBL Biomedical Laboratories). Untreated cells were taken as the control group (vehicle, *n* = 8). After incubation, cells were lysed in 600 μl of RLT lysis buffer (Qiagen, RNeasy Plus Mini Kit). RNA isolation and purification were carried out as described by the manufacturer (Qiagen, RNeasy Plus Mini Kit). For homogenisation, cell lysates were passed through QIAshredder spin columns (Qiagen) and the flow-throughs were then transferred to gDNA eliminator spin columns. RNAs were purified on RNeasy spin columns and eluted with 40 μl of RNase-free water. Total RNAs were quantified with the Quant-IT RNA BR assay-500 and a Qubit Fluorometer (Invitrogen). The quality of RNAs (500 ng) was checked on a 1.5% agarose gel.

### Blood retrieval and RNA extraction and qualification

A volume of 2.5 ml of whole blood sample from each patient was retrieved in PAXgene blood RNA tubes 4 weeks prior to antiviral therapy and every 2 weeks during the 12 weeks after onset of treatment, stored at − 20 °C and then transferred to − 80 °C. PAXgene™ Blood RNA tubes (PreAnalytix) contain a reagent that protects RNA molecules from degradation by RNAses and ex vivo changes in gene expression. Samples were distributed randomly in the different sets of extractions. Total RNA from the 2.5 ml of whole blood was extracted using the PAXgeneTM Blood RNA Kit (ref 28704, PreAnalytix). Extracted RNA was isolated using a Qiagen QIAcube system, following the manufacturer’s protocol for PAXgene Blood RNA part 1, automated protocol. During sample preparation and RNA extraction, standard precautions were taken to avoid RNA degradation by RNAses. Total RNA concentrations were determined with a Qubit Fluorometer (Invitrogen) and the Quant-IT RNA BR assay (Invitrogen).

### Reverse transcription and quantitative real-time PCR

Reverse transcription was carried out on 12.5 ng/μl of RNA per sample using the Takara Kit (PrimeScript RT, Takara, ref no. RR037A). The resulting cDNA was combined with TaqMan universal PCR Master Mix (Applied Biosystems, ref no. 4369016) and with the following specific gene probes: ADAR1-1 (ADAR1a-p150) (Hs01020780), ADAR1-5 (ADAR1-b-p110) (Hs01017596), ADAR2 (Hs00210562), PDE8A (Hs00400174), GAPDH (Hs02758991), HPRT1 (Hs02800695), TBP (HS00427620) and PGK1 (Hs99999906) (Applied Biosystems, from Life Technologies) in 20 μl final volume. Quantitative PCR was performed in 384-well plates on LightCycler 480 Real Time PCR instrument (Roche). The analysis was performed using a second derivative absolute quantification, normalised by the geometric mean of four housekeeping genes (HPRT1, GAPDH, TBP and PGK1).

### NGS library preparation

For next-generation sequencing (NGS) library preparation, a targeted approach was employed in order to selectively sequence the region of interest within intron 9 of the PDE8A gene (Supplementary Figure 1). Validated PCR primers were used to amplify the region of interest by PCR (Table 2). For PCR amplification, the Q5 Hot Start High Fidelity enzyme (New England Biolabs) was used according to the manufacturer’s guidelines (M0494S). The PCR reaction was performed on a PeqSTAR 96x thermocycler using optimised PCR protocol. Both quantity and quality of the PCR product were assessed using a LabChip Gx instrument (PerkinElmer). Purity of the amplicon was determined and quantification was performed using the fluorescence-based Qubit method. After quality control, the PCR reactions were purified using magnetic beads (HighPrep PCR MAGbio system, Mokascience). DNA was then quantified using the Qubit system and purification yield was calculated. Next, samples were individually indexed by PCR amplification using Q5 Hot start High fidelity PCR enzyme (New England Biolabs) and the Illumina 96 Indexes kit (Nextera XT index kit; Illumina). Samples were then pooled into a library and purified using the Magbio PCR clean-up system. The library was denatured and loaded onto a sequencing cartridge according to Illumina’s guidelines for sequencing FASTQ only on a MiSeq platform. A commercial total RNA pool from human blood peripheral leukocytes (Clontech, no. 636592) was incorporated into the libraries to determine variability between different sequencing flow cells during the course of the experiment. NGS libraries were sequenced (single-read sequencing, read length 225 bp) at standard concentrations and spiked in to introduce library diversity using PhiX Control V3 (Illumina). Experiments were performed five times independently.

**Table 2:** Names and sequences of PCR primers used to measure PDE8A mRNA editing by a next-generation sequencing (NGS)–based method. A sequencing library containing multiple samples was generated using a 2-step PCR library preparation protocol. The library was sequenced on a next-generation sequencing system (MiSeq, Illumina)

Primer name	Forward primer sequence	Length
PDE8A-Seq2-forward	ATGCAAGTTGTGGACATGGAG	21
PDE8A-Seq3-reverse	TTCTGAAAACAATGGGCACCA	21

### Bioinformatics analysis of sequencing data

The sequencing data was downloaded from the Miseq sequencer (Illumina) as FASTQ file. To evaluate sequencing quality, an initial quality of each raw FASTQ file was performed using FastQC software (version 0.11.3). A minimal sequencing depth of 50,000 reads for each sample was considered for further analysis. A pre-treatment step was performed consisting of removing adapter sequences and filtering of the sequences according to their size and quality score. All short reads (< 100 nts) and reads with average QC < 30 were removed. To improve sequence alignment quality of the sequences, flexible read trimming and filtering tools for Illumina NGS data were used (fastx_toolkit v0.0.14 and PRINSEQ version 0.20.4). After pre-processing steps were performed, an additional quality control of each cleaned FASTQ file was carried out prior to further sequence processing.

Alignment of the processed reads was performed using bowtie2 (version 2.2.5) with end-to-end sensitive mode. The alignment was done to the latest annotation of the human genome sequence (GRCh38) and reads multiple alignment regions, reads with poor alignment quality (Q < 30) or reads containing insertion/deletion (INDEL) were taken out of the further analysis. Filtering of file alignment was carried out with SAMtools software (version 1.3.1) that provide various utilities for manipulating alignments in the SAM format, including sorting, merging, indexing and generating alignments in a per-position format.

Next, SAMtools mpileup was used to pileup obtained alignment results data from multiple samples simultaneously. An in-house script was run to count the number of different ATGC nucleotides in each genomic location (‘base count’). So, for each genomic location, the home-made script computes the percentage of reads that have a ‘G’ [Number of ‘G’ reads/(Number of ‘G’ reads + Number of ‘A’ reads) × 100]. The genomic location ‘A’ reference with percentage in ‘G’ reads > 0.1 is automatically detected by the script and is considered as ‘A-to-I edition site’. The last stage was to compute the percentage of all possible isoforms of PDE8A transcripts. By definition, the relative proportion of RNA editing at a given editing ‘site’ represents the sum of editing modifications measured at this unique genomic coordinate. Conversely, an mRNA isoform is a unique molecule that may or may not contain multiple editing modifications on the same transcript. For example, the PDE8A mRNA isoform BC contains a modification on both site B and site C within the same transcript.

### Statistical analysis of data

All statistics and figures were computed with the ‘R/Bioconductor’ statistical open-source software and GraphPad Prism software (version 7.0) (Gentleman et al. 2004). Biomarker (i.e. RNA editing sites and isoforms of PDE8A and mRNA expression of ADARs) values are usually presented as mean ± standard error of the mean (SEM). A differential analysis was carried out using the Mann-Whitney test and a *p* value below 0.05 was considered as statistically significant. The accuracy of each biomarker and its discriminatory power was evaluated using a receiver operating characteristic (ROC) analysis. ROC curves are the graphical visualisation of the reciprocal relation between the sensitivity (Se) and the specificity (Sp) of a test for various values.

In addition, all biomarkers were combined with each other to evaluate the potential increase in sensibility and specificity using an mROC multivariate approaches (Kramar et al. 2001). mROC is a dedicated programme to identify the linear combination (Su and Liu 1993), which maximises the AUC (area under the curve) ROC (Staack et al. 2006). The equation for the respective combination is provided and can be used as a new virtual marker *Z* as follows:

$$ Z=a\times \mathrm{bmk}1+b\times \mathrm{bmk}2+c\times \mathrm{bmk}3, $$

where *a*, *b* and *c* are the calculated coefficients and biomarkers (bmk) 1, 2 and 3 are the levels of the biomarker.

Principal component analysis (PCA) was performed using scatterplot3d (v0.3-40) R package. PCA involves a mathematical procedure that represents the maximum of the data information by reducing the space dimension.

## Results

### Clinical trial design

In this study, we took advantage of the well-documented and well-characterised mood alterations observed in hepatitis C–infected patients undergoing antiviral therapy with IFN and ribavirin. A small cohort of ten individuals with hepatitis C virus never treated with interferon and ribavirin and without prior records for psychiatric disorders was recruited over different medical hospitals in France. At inclusion, patients underwent harmonised psychiatric assessment and at repeated interval during the course of treatment. In this particular setting, every patient acts as its own control and evolution of the patient can be monitored over time. The general descriptive characteristics of the cohort are given in Table 1 as well as the study design.

### Assay and sequence description in white blood cells

**Fig. 1:**
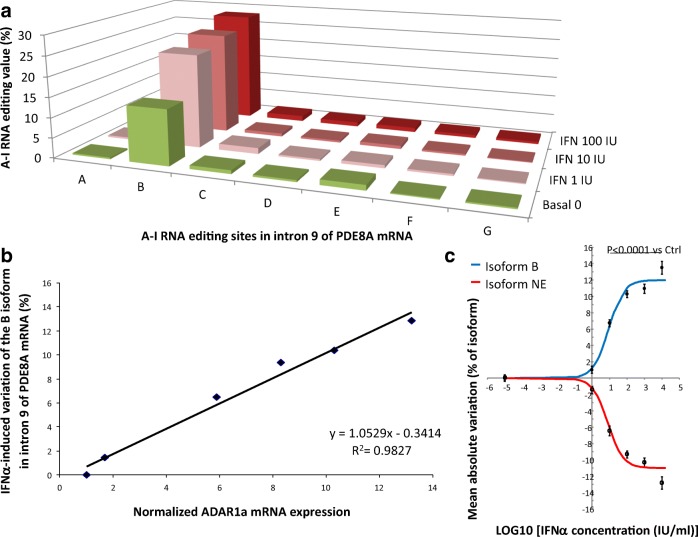
IFN-α treatment induced alterations of the relative proportion of PDE8A RNA editing sites in SH-SY5Y cell line. **a** NGS-based quantification of RNA editing on intron 9 of the PDE8A gene in SH-SY5Y cell culture samples for sites A–G. **b** Correlation, in SH-SY5Y cells, between the relative quantities (RQ) of ADAR1-1 (ADAR1a, p150) transcript level induced by increased concentrations of IFN-α and the relative increase in the proportions of isoform B in the editing profile of PDE8A RNA. The isoform B is defined as the isoform in which the edited site B is alone under the edited form. **c** Relationship between applied IFN-α concentrations and the mean Δ of variation of the B and non-edited (NE) isoforms in the SH-SY5Y cells. Each point represents the mean ± SEM (*n* = 8) of the individual values measured 48 h after administration in the incubating medium of 0, 1, 10, 100, 1000 and 10,000 IU of IFN-α

As we previously identified IFN-induced ADAR1-1 (ADAR1a, p150) induction and RNA editing events on the 5-HT2cR in the neuroblastoma cell line SH-SY5Y by capillary electrophoresis single-strand conformation polymorphism (CE-SSCP) (Cavarec et al. 2013), we first investigated IFN-induced RNA editing on the PDE8A gene in the same cell line. We thus collected total RNA from a human neuroblastoma cell line (SH-SY5Y) cultured in presence of interferon for 48 h and compared with vehicle control. To measure RNA editing by NGS, we employed a targeted sequencing approach, which allows to detect very rare events (threshold of 0.1% editing rate). Sample sequencing was performed with an average of at least 50,000 sequences per sample (ultra-deep sequencing). With this technique, we show that virtually all adenosines in the amplified region sequenced were subjected to RNA editing. In total, NGS sequencing identified up to 27 adenosines which were identified to be actively edited by A-to-I RNA editing above a threshold of 0.1% in at least one experiment, suggesting that most ADAR activity takes place in cluster. Nevertheless, we only considered for further analysis sites A to G, all of which have been observed to be edited with a cutoff > 0.1% in all experiments (Fig. 1a and Supplementary Figure 1). Both in basal and in IFN-treated cells, the previously identified B site was found to be most actively edited in SH-SY5Y cells, with a relative proportion on 13.8% of editing events on this site. Treatment of IFN resulted in doubling of the RNA editing at this site reaching up to 28% of the total PDE8A mRNA (Fig. 1a). Additionally, we observed that the increase in the level of RNA editing of the B site was correlated with the relative quantity of ADAR1-1 (ADAR1a, p150) transcript as measured by qPCR (*R*^2^ = 0.9827) (Fig. 1b). More importantly, we show that the IFN-dependent increase of RNA editing on the B site of PDE8A was concomitant to a decrease of the relative proportion on the non-edited mRNA (Fig. 1c), suggesting that increased ADAR1-1 (ADAR1a, p150) expression led to increased editing from the non-edited (NE) isoform. In sum, while we confirmed IFN-dependent A-to-I editing on the PDE8A B gene, we further comprehensively described the RNA editing events that occur on intron 9 of the PDE8A gene in the human neuroblastoma cell line (Supplementary Figure 1).

**Fig. 2:**
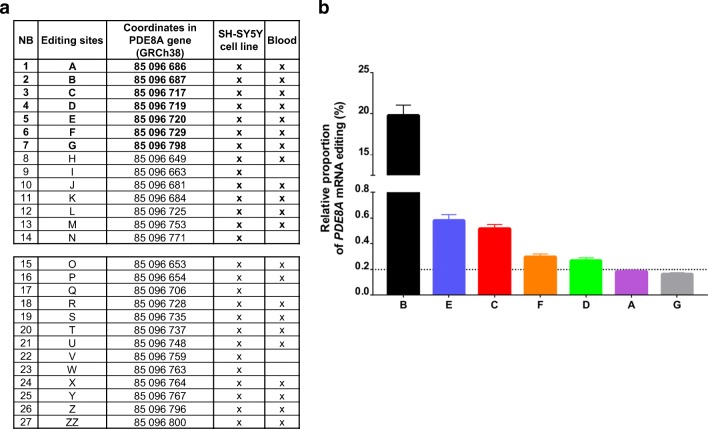
Listing of RNA editing sites in PDE8A region of interest in SH-SY5Y cell line and human blood cells. **a** Comparison of the RNA editing sites identified in the human neuroblastoma cell line SH-SY5Y and in human blood cells. X indicates when the editing site is measured at least in one experiment/patient with a cutoff > 0.1%. **b** Relative proportion of PDE8A mRNA editing at all sites that display more than 0.1% editing. Histograms show mean values ± SEM (*n* = 5)

Next, we compared the RNA editing activity in the SH-SY5Y human cell line with that in the RNA extracted from patients’ blood. While most sites were found to be common to both cell types, such as the B site, small variations could be detected (Fig. 2a). Among these, 5 editing sites that were identified in SH-SY5Y cells could not be detected in white blood cells (WBC). Indeed, despite ultra-deep sequencing of the region, A-to-I RNA editing events could not be detected on sites I, N, Q, V and W in human blood cells (Fig. 2a). By means of our powerful sequencing approach, a clear RNA editing profile was identified in human WBC. As previously observed by Orlowski and colleagues, the B site was the most edited site in the region found while E, C, F, D, A and G sites were edited with a frequency of at least 0.1% in all patients (Fig. 2b).

### Longitudinal analysis of RNA editing and ADAR enzymes in blood

**Fig. 3:**
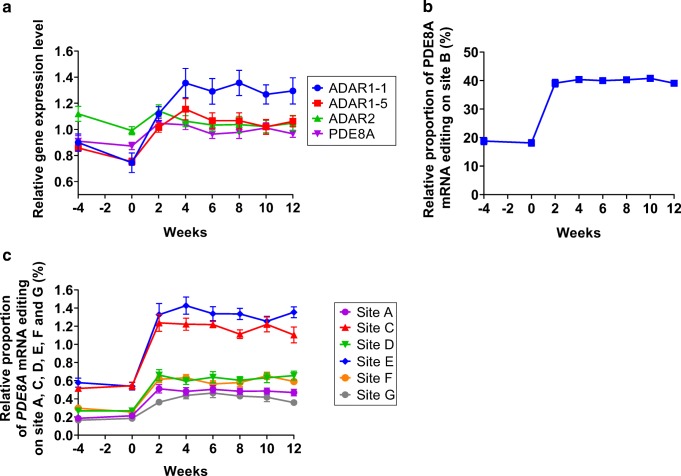
Longitudinal analysis of ADAR enzymes gene expression and PDE8A gene expression and editing in HCV patients over the course of IFN treatment. **a** Longitudinal analysis of ADAR1-1 (ADAR1a, p150), ADAR1-5 (ADAR1b, p110), ADAR2 and PDE8A gene expression of all HCV patients (*n* = 10). **b** Longitudinal analysis of RNA editing on site B of the PDE8A gene of all HCV patients (*n* = 10). **c** Longitudinal analysis of RNA editing on the other identified main sites of the PDE8A gene of all HCV patients (*n* = 10). The mean values are displayed ± SEM (*n* = 10)

Transcript levels of the family of ADAR and PDE8A enzymes were analysed at different time points before and during antiviral treatment. While significant changes in gene expression were not observed for PDE8A and ADAR2 transcripts, a significant upregulation of ADAR1-1 (ADAR1a, p150) and ADAR1-5 (ADAR1b, p110) transcript level was observed as early as 2 weeks after onset of therapy (Fig. 3a). Indeed, transcript level of ADAR1-1 (ADAR1a, p150) almost doubled during the course of treatment and reached plateau 4 weeks after treatment onset (Fig. 3a). ADAR1-5 (ADAR1b, p110) displayed a very similar gene expression profile as compared with ADAR1-1 (ADAR1a, p150), though with a less marked increase. The similar expression profile that was observed between ADAR1-1 (ADAR1a, p150) and ADAR1-5 (ADAR1b, p110) suggests that ADAR1 gene expression is uniquely altered by antiviral treatment as opposed to ADAR2 and PDE8A expression that showed no significant differences during the course of the treatment. Next, by applying the ultra-deep sequencing approach, the RNA editing profile of the PDE8A gene was analysed. Interestingly, editing on the B site of the PDE8A transcript doubled within 2 weeks and reached 40% of total PDE8A reads sequenced (Fig. 3b). More strikingly, the increased expression of ADAR1-1 (ADAR1a, p150) paralleled that of increased editing in PDE8A site B (Fig. 3a, b). Remarkably, the RNA editing increase of the PDE8A transcript was highly homogenous over the whole studied population as indicated by the very small standard errors of the mean. Overall, considering a threshold of 0.1%, six additional sites were analysed. While minor variations were observed on sites D, E, F and G, we observed a significant increase in editing values for sites E and C upon treatment, which mimicked the one observed for site B. Interestingly, the overall increase of RNA editing on all edited sites following antiviral therapy reached a plateau level between 2 and 4 weeks after therapy onset (Fig. 3c). Consistent with data obtained in SH-SY5Y, IFN injection in hepatitis C–infected patients induced a rapid and strong increase in RNA editing activity in the patients’ blood cells.

### Predictive algorithm to detect patients with increased risk to have mood alterations

**Fig. 4:**
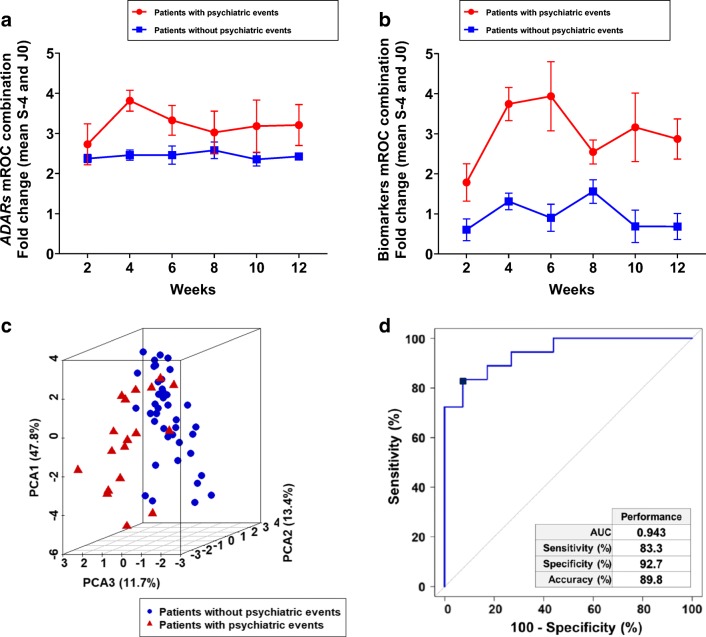
Diagnostic performances of a combination of biomarkers. **a** Longitudinal analysis of an mROC combination of biomarkers composed of ADAR gene expression in the group of patients that showed clinical depression (*n* = 3) and the group that did not show clinical depression (*n* = 7). **b** Longitudinal analysis of an mROC combination of ADAR gene expression and RNA editing biomarkers in the group of patients that showed clinical depression (*n* = 3) and the group that did not show clinical depression (*n* = 7). **c** Principal component analysis of ADARs, PDE8A gene expression and PDE8A mRNA editing sites in the group of patients that showed clinical depression (*n* = 3) and the group that did not show clinical depression (*n* = 7). The scatter plot visualises the first, second and third principal components and respective variance percentages on the *x*-, *y*- and *z*-axis of ratio (fold change) of biomarkers before and after treatment in longitudinal analysis. Red triangle: patients with psychiatric events. Blue circle: patients without psychiatric events. **d** ROC curve of a combination of biomarkers to separate the population with and without psychiatric disorders (depression). Diagnostic performances of the algorithm are indicated

Different clinical evaluations have been performed in IFN/ribavirin treated patients, including MADRS, YMRS, MATHys and emotional reactivity during treatment. By dividing the population into two groups composed of patients having a psychiatric event (combined clinical evaluations) or not, we analysed the combination of ADAR gene expression. We obtained a marked difference in ADAR1-1 (ADAR1a, p150) and ADAR1-5 (ADAR1b, p110) gene expression levels between the groups with and without psychiatric events (Fig. 4a). Interestingly, a similar separation was also observed by analysing the combination of ADAR gene expression and RNA editing of PDE8A transcript (Fig. 4b). While gene expression peaked at 4 weeks after the onset of antiviral therapy in the patients experiencing mood disorders, combining RNA editing sites leads to a clear induction as long as 6 weeks after treatment in the group that displayed a psychiatric event (Fig. 4b). By combining both gene expression data and RNA editing events, a strong and marked differentiation was obtained between both groups by principal component analysis (Fig. 4c). Diagnostic performances of the combination of biomarkers were assessed by ROC analysis (Fig. 4d). Combining RNA editing and specific gene expression biomarkers clearly showed high specificity and sensitivity in identifying IFN-treated patients who experienced a psychiatric event during the course of the treatment. Finally, by applying an mROC algorithm to all patients at all time points, we tested the combined biomarkers to identify at risk patients. While the combination of biomarkers was highly robust over the whole course of the pilot study to detect the three patients that developed psychiatric events, 3 out of the 18 calls (16.7%) corresponding to a specificity of 83.3% (see Fig. 4d) were false negatives. On the other hand, only 3 out of the 41 calls in the group of patients that did not show clinical signs of depression were false positives (7.3%) corresponding to a sensitivity of 92.7% (see Fig. 4d). Overall, identified blood biomarkers allow accurate and robust detection of patients that are at risk to develop a depression in the context of a pharmacological treatment such as IFN-α and ribavirin.

## Discussion

In this study, we have identified RNA editing blood biomarkers to detect HCV patients at risk to develop treatment-emergent depression during the course of IFN-α and ribavirin therapy. Clinical evaluation of HCV patients using MADRS score was used prior to onset and during the course of IFN-α and ribavirin treatment to track depressive episodes. In parallel, 80 blood samples were collected for analysis of ADAR and PDE8A gene expression and targeted quantification of RNA editing events in WBC. Next, based on clinical assessments, the study population was divided into a group that showed evidence for treatment-emergent depression and a second group that did not. Interestingly, we observed that IFN-α treatment led to an increase in ADAR1-1 (ADAR1a-p150) and ADAR1-5 (ADAR1b, p110) gene expression as well as a significant increase of RNA editing on the B site of the intron 9 of the PDE8A mRNA. By combining the biomarkers, we generated an algorithm for detecting patients at risk to develop a treatment-emergent psychiatric event. Since depression is one of the most important complications during antiviral treatment with interferon and ribavirin, the assay may be valuable to support patient monitoring.

IFN-α-induced depression is one of the most common psychiatric complications in HCV patients who receive IFN-α and ribavirin treatment. Up to 30% of HCV patients develop IFN-induced depression within the first 3 months (Schafer et al. 2007; Su et al. 2010; Udina et al. 2012). Results from the current study are in line with the prevalence rate of psychiatric side effects with 30% of HCV patients that developed depression during the 12 weeks of IFN-α and ribavirin therapy.

In the current longitudinal study, we show that high acute doses of IFN-α induced a very marked increase in ADAR1-1 (ADAR1a-p150) expression as well as alterations in the RNA editing activity on the PDE8A gene. Unexpectedly, both ADAR1-1 (ADAR1a-p150) and ADAR1-5 (ADAR1b-p110) displayed an increase in gene expression while ADAR2 did not show alteration in transcript level. While an increase in ADAR1-1 (ADAR1a-p150) expression upon IFN-α treatment has been well documented, this is unexpected and has not yet been clearly described for ADAR1-5 (ADAR1b, p110) (George and Samuel 1999). In light of these differences, it seems likely that the observed changes in RNA editing profile upon antiviral therapy are the consequence of the changes in ADAR1-1 (ADAR1a-p150) and ADAR1-5 (ADAR1a-p150) activity. As observed for the RNA editing level on the B site of the PDE8A intron 9, the RNA editing activity response to IFN-α was strong and paralleled that of ADAR1-1 (ADAR1a-p150) and ADAR1-5 (ADAR1b-p110). Of note, we cannot exclude a contribution of ribavirin on the effect measured both on gene expression and on RNA editing activity, although we previously showed that in human neuroblastoma cell line SH-SY5Y, only IFN-α treatment but not ribavirin alone resulted in increased gene expression of ADAR1-1 (ADAR1a-p150) and RNA editing of 5-HT2cR (data not shown).

Our study has a number of limitations. First, the current study was performed in a relatively small number of enrolled patients (*n* = 10) and could be confirmed in a larger population. However, despite the small size of patients, the results are representative of the prevalence of depression observed in the general population; we followed up patients during 16 weeks and then ran analysis on 80 blood samples. Second, this study shows high predictive performance using RNA editing activity of PDE8A. The identification of novel RNA editing targets is currently ongoing and could significantly improve the performance of the test.

Recently, a large whole transcriptome study showed a significant association between major depressive disorder (MDD) and type I interferon gene expression. Interestingly, ADAR gene expression was significantly induced in white blood cells of MDD patients when compared with healthy controls (Mostafavi et al. 2014). Elevated interferon levels, as observed in MDD, activate the innate immune system and regulate expression of genes that interfere with viral replication. This finding could not be explained by confounding medication use or diseases such as virus. However, in this context, it is not surprising that psychiatric comorbidity is high in HCV patients. Interestingly, psychiatric comorbidity also depends on the type of viral infection since depression is reported at much higher rate for HCV than for hepatitis B virus infection (Weissenborn et al. 2006). The underlying mechanistic explanation for this difference between the two types of viral infection still remains to be addressed and understood.

HCV belongs to the Flaviviridae family which includes neurotropic viruses among which encephalitis viruses, dengue and others. In line with this classification, HCV was also observed to invade the central nervous system. Post-mortem analysis of HCV-infected patients identified macrophages/microglia cells as harbouring HCV (Radkowski et al. 2002). Since HCV can also replicate in peripheral blood mononuclear cells (PBMC), the hypothetical route for CNS infection could be provided by these cells since under certain condition, these cells can enter the brain. Susceptibility to HCV neuroinvasion could potentially underlie and embody the likelihood of an HCV-infected patient to develop depression. In line with this hypothesis, inflammatory signalling is known to induce alteration in blood-brain barrier functioning and permeability (for review, see Liebner et al. 2018). The use of IFN-α as antiviral therapy is now being replaced by a new generation of direct-acting antiviral agents whose costs are extremely high. However, access to such medications remains a serious challenge in developing countries, and interferon and ribavirin combination is still in use in many countries due to the high costs of new treatments (Kamal 2018).

The recent technological shift brought about by next-generation sequencing has opened the way to innovative high-throughput drug screening solutions that can quantitatively measure modifications on RNA to detect, for instance, candidate drugs having a risk to induce suicidal ideation in later stages of development. Quantification of RNA editing as dynamic and mechanism-related biomarker offers unprecedented potential. In this study, we have shown that RNA editing biomarkers can be used for monitoring depression state in HCV patients during antiviral therapy. The use of RNA editing biomarkers, from prediction of adverse drug reactions in early preclinical phase to stratification of patients or companion tests in later stages, opens avenue to precision medicine in psychiatry.

## Electronic supplementary material

Supplementary Figure 1 Sequence of the PDE8A region of interest and all annotated RNA editing sites. Positions on Chromosome 15 refer to Genome Reference Consortium Human Build 38 (GRCh38). The forward and reverse primers used for preparation of the sequencing library are shown. (PNG 282 kb)

High Resolution (TIF 3323 kb)

## Abbreviations

ADAR: adenosine deaminase acting on RNA
HCV: hepatitis C virus
IFN-α: interferon alpha
MDD: major depressive disorder
NGS: next-generation sequencing
PDE8A: phosphodiesterase 8A
WBC: white blood cells
