# Supplementary figures and images for: RNA editing blood biomarkers for predicting mood alterations in HCV patients
Source: J Neurovirol. 2019 Jul 22;25(6):825–36. doi: 10.1007/s13365-019-00772-9 (PMC6920238; doi:10.1007/s13365-019-00772-9)

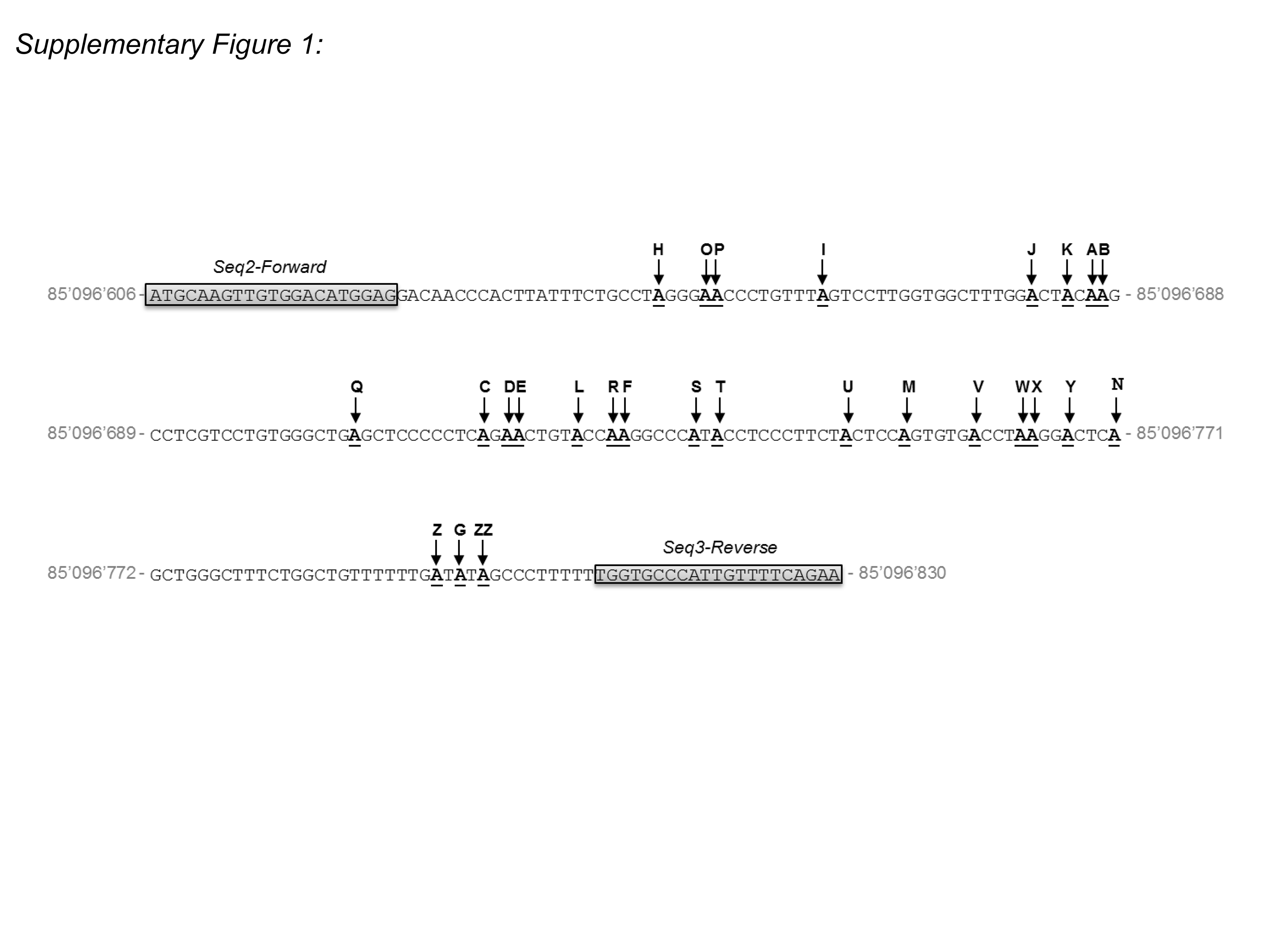

Supplement: Supplementary file 1 — Sequence of the PDE8A region of interest and all annotated RNA editing sites. Positions on Chromosome 15 refer to Genome Reference Consortium Human Build 38 (GRCh38). The forward and reverse primers used for preparation of the sequencing library are shown. (PNG 282 kb) [file 13365_2019_772_Fig5_ESM.png]
